# The larvae of *Sericostoma bergeri* Malicky, 1973 and *Sericostoma herakles* Malicky, 1999 (Trichoptera, Sericostomatidae)

**DOI:** 10.3897/zookeys.695.14531

**Published:** 2017-09-05

**Authors:** Johann Waringer, Hans Malicky

**Affiliations:** 1 Department of Limnology and Bio-Oceanography, University of Vienna, Althanstrasse 14, A-1090 Vienna, Austria; 2 Sonnengasse 13, A- 3293 Lunz am See, Austria

**Keywords:** Description, distribution, larva, identification, West Palearctic fauna

## Abstract

This paper describes the previously unknown larvae of *Sericostoma bergeri* and *S. herakles* (Trichoptera: Sericostomatidae) restricted to European Ecoregion 6 (= Hellenic western Balkan). Information on the morphology of the larvae is given, and the most important diagnostic features are illustrated. *Sericostoma bergeri* and *S. herakles* can be easily separated from known sericostomatid larvae of Ecoregion 6 (*Schizopelex huettingeri*, *Oecismus monedula*, *Sericostoma flavicorne* and *S. personatum*) by the shape of the pronotum, presence or lack of a comma-like marking on the lateral protuberance, by the number of setae on abdominal dorsum IX, and by distribution patterns. With respect to the latter, *S. bergeri* is a micro-endemic of the Greek Islands of Euboea and Andros whereas *S. herakles* is an endemic of the Peloponnese. The species are integrated in a dichotomous key including the currently known Sericostomatidae larvae of the Hellenic western Balkan. In addition, ecological information on the two species is provided.

## Introduction

From Europe, 18 species of genus *Sericostoma* Latreille, 1825 are known (Graf et al. 2008; Malicky 2004, 2005a, b, 2014), with four species also present in European Ecoregion 6 (= Hellenic western Balkan). From the latter, only *Sericostoma flavicorne* Schneider, 1845 and *Sericostoma personatum* (Kirby & Spence, 1826) were described in the larval stage to date (Pitsch 1993). Several years ago, however, Hans Malicky managed to collect larvae and adults of the two remaining *Sericostoma* species of Ecoregion 6: *S. bergeri* Malicky, 1973 from the Greek islands of Euboea and Andros and *S. herakles* Malicky, 1999 from the Peloponnese. This material enabled us to infer reliable diagnostic characters for the larval description and to use this information for integrating the two species in the key of the previously known Sericostomatidae larvae of the Hellenic western Balkan provided by Karaouzas and Waringer (2017).

## Material and methods

Three final instar larvae and numerous adults of *Sericostoma bergeri* were sampled by H. Malicky on the Greek island of Andros at Refmata (37°52'N, 24°50'E, 220 m a.s.l.) on 21 October 1980. In addition, one final instar larva and numerous adults of *S. herakles* were obtained by the same collector at Kefalarion, Peloponnese, Greece (37°54'N, 22°31'E, 670 m a.s.l.) on 19 May 1974. Immature stages were picked from the mineral substrate with forceps, adults were collected using light traps, and the material was preserved in 70% ethanol. The larvae were studied and photographed using a Nikon SMZ 1500 binocular microscope with DS-Fi1 camera and NIS-elements D 3.1 image stacking software for combining 6–46 frames in one focused image. Species association was enabled by the fact that both larvae and adults were collected at the same locations where other Sericostomatidae were lacking.

**Deposition of voucher specimens**: Final instar larvae of *Sericostoma bergeri* and *S. herakles* are deposited in the collections of the authors in Lunz am See and Vienna. Comparative material of *Schizopelex huettingeri* Malicky, 1974 (3 final instar larvae), *Oecismus monedula* (Hagen, 1859) (1 final instar larva) and *Sericostoma personatum* (Kirby & Spence, 1826) / *Sericostoma flavicorne* Schneider 1845 (15 final instar larvae) are deposited in the collection of J. Waringer (Vienna, Austria). We used the morphological terminology of Wiggins (1998), Pitsch (1993), and Waringer and Graf (2011).

## Results

### Sericostoma bergeri

Taxon classificationAnimaliaTrichopteraSericostomatidae

Malicky 1973

#### Description of the 5^th^ instar larva.

**Diagnosis.** Pronotum with convex ventral border; anterolateral pronotal corner short and knob-like; with black comma-like marking on lateral protuberance; setal counts on abdominal dorsum IX 18–41.

**Biometry.** Body length of 5th instar larvae ranging from 16.0 to 17.5 mm, head width from 1.69 to 1.76 mm (n = 3).

**Figures 1–5.**
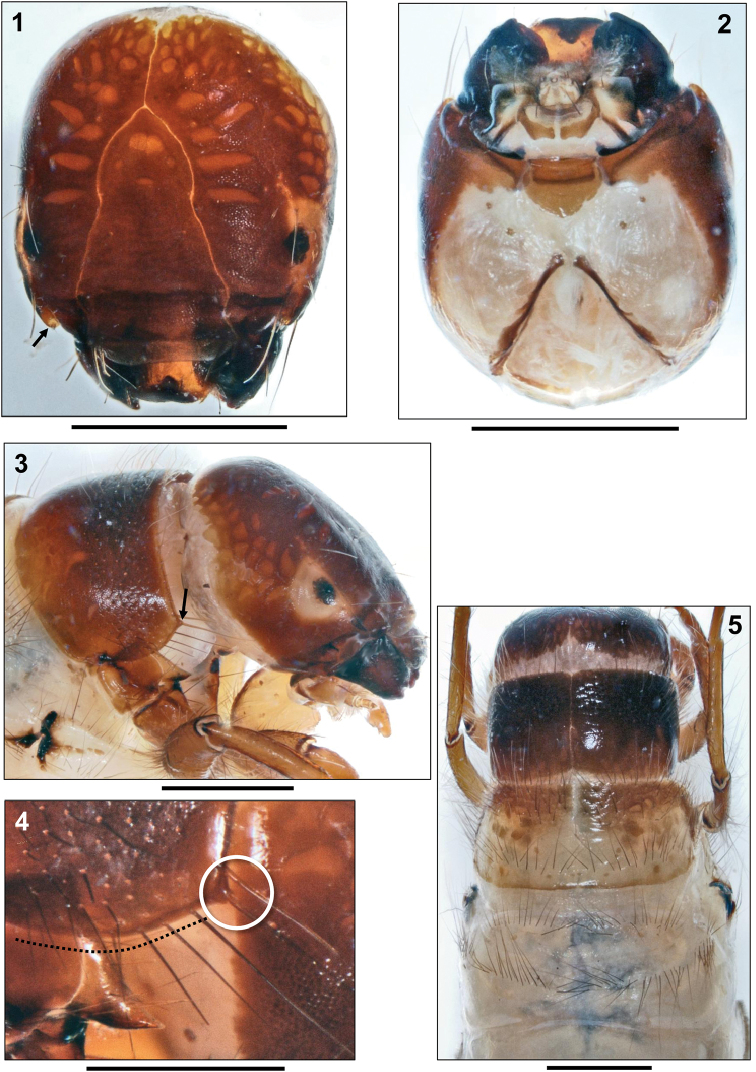
*Sericostoma bergeri* Malicky, 1973, final instar larva. **1** Head, dorsal view (arrow: antenna) **2** Head, ventral view **3** Head and pronotum, right lateral view (arrow: anterolateral corner of pronotum) **4** Detail of pronotum (right lateral) showing small and knoblike anterolateral corner (white circle) and curved ventral outline (black dots) **5** Head, thorax and abdominal segment I, dorsal. Scale bars: 1 mm.

**Head**. Head capsule roundish, dorsally medium to orange brown, posterolaterally and ventrally whitish; with slightly granulated surface and large, elongated, orange muscle attachment spots (Figs 1–3). Distinct whitish spade-like patch present around each eye (Fig. 3). With ridge extending from posterodorsal corner of white spade-like ring around each eye (Fig. 3) to anterior parietal margin where ridge creates an inwardly-bending groove bearing the antenna (Fig. 1, arrow). Frontoclypeus with shallow central constriction; a shallow secondary constriction situated subapically near anterior border (Fig. 1). Head capsule with complete set of 18 pairs of primary setae: 10 dorsal and 2 ventral primary setae on each parietal, 6 pairs of primary setae on frontoclypeus. Labrum medium brown, narrowly rectangular, with 6 pairs of primary setae. Submentum separating the genae incompletely, broadly shield-shaped, light brown, with darker brown rectangular anterior sclerotization (Fig. 2). Mandibles blackish brown, of shredder type, with 4 terminal teeth (Fig. 2).

**Figures 6–11.**
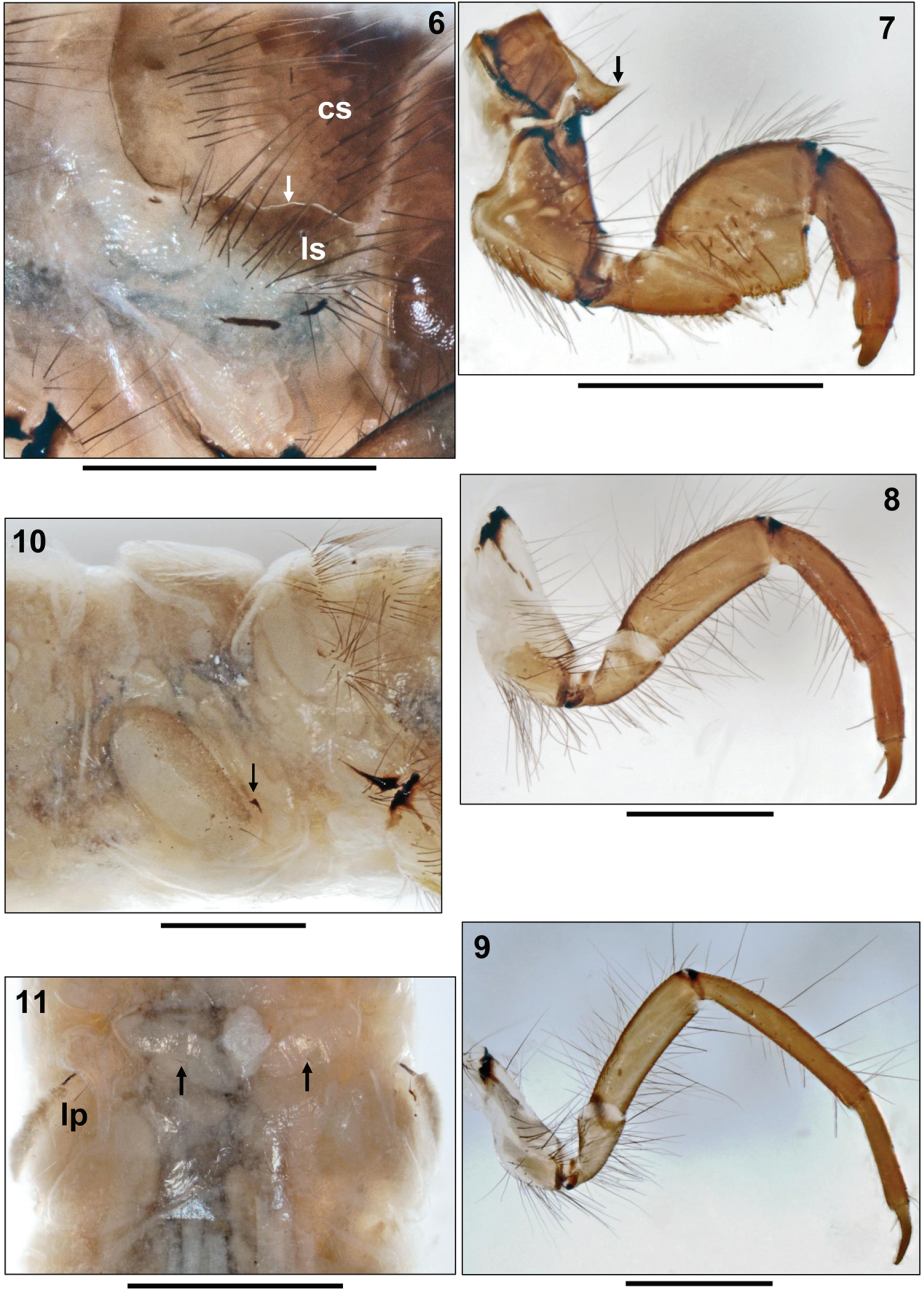
*Sericostoma bergeri* Malicky, 1973, final instar larva. **6** Right anterolateral section of mesonotum (arrow: suture between central and lateral mesonotal sclerite; cs: central sclerite; ls: lateral sclerite) **7** Right foreleg, posterior face (arrow: propleuron, anterior process) **8** Right midleg, posterior face **9** Right hind leg, posterior face **10** Metanotum and abdominal segment I, right lateral (arrow: lateral protuberance with black, comma-like marking dorsally of lateral protuberance seta) **11** Abdominal sternum I (arrows: single sa1 setae; lp: lateral protuberance seta). Scale bars: 1 mm.

**Thorax.** Pronotum dark brown (Fig. 5), in some specimens slightly paler on posterior half (Fig. 3). Without transverse ridge present in other caddisfly taxa (e.g., Limnephilinae), heavily sclerotized, with anterolateral corner creating a tiny, knob-like projection (Fig. 4, white circle). The two pronotal plates mesially meeting in a narrow, straight suture; surface smooth (Fig. 5). Ventral pronotal margin curved (Fig. 4, dotted line). Each pronotal half covered by 223–235 setae concentrated on anterior pronotal section. Anterior pronotal margin with row of pale, curved setae (Fig. 3). Anterior process of propleuron long and corniform (Fig. 7, arrow). Mesodorsum covered by 4 sclerotized plates (2 large central, 2 small lateral), anterior border medium brown, with oval, pale muscle attachment spots, posterior section whitish, with brown muscle attachment spots (Fig. 5); suture between central and lateral plates inconspicuous (Fig. 6, arrows). Setal counts per central sclerite are 64–72 in anterior group and 25–30 in posterior group; lateral sclerite with 49–57 dark setae (Fig. 6).

Prosternal sclerites and prosternal horn lacking. Metadorsum covered by colourless and barely visible weak sclerites arranged in 2 parallel transverse bands. Setal counts per sclerite are 27–35 setae in anterior group, 40–46 setae in posterior group (Fig. 5).

Legs medium to light brown (Figs 7–9). Foreleg short and stout, femur distally enlarged and flattened, thereby creating an edge interacting with tibia when bent inwards (Fig. 7). Coxa with ventral group of long black setae, trochanter with dense ventral brush of pale, flexible setae. Dorsal edge of femur with large groups of dark setae. Tibia with groups of long dark dorsal and ventral setae and with pale apical spine. Strong tarsal claw sickle-shaped, with stout pale basal spine. Midleg much more slender, coxa weakly sclerotized, femur not enlarged. Hind leg even more slender, tarsal claw elongated, setation less than in other legs (Figs 7–9).

**Figures 12–19.**
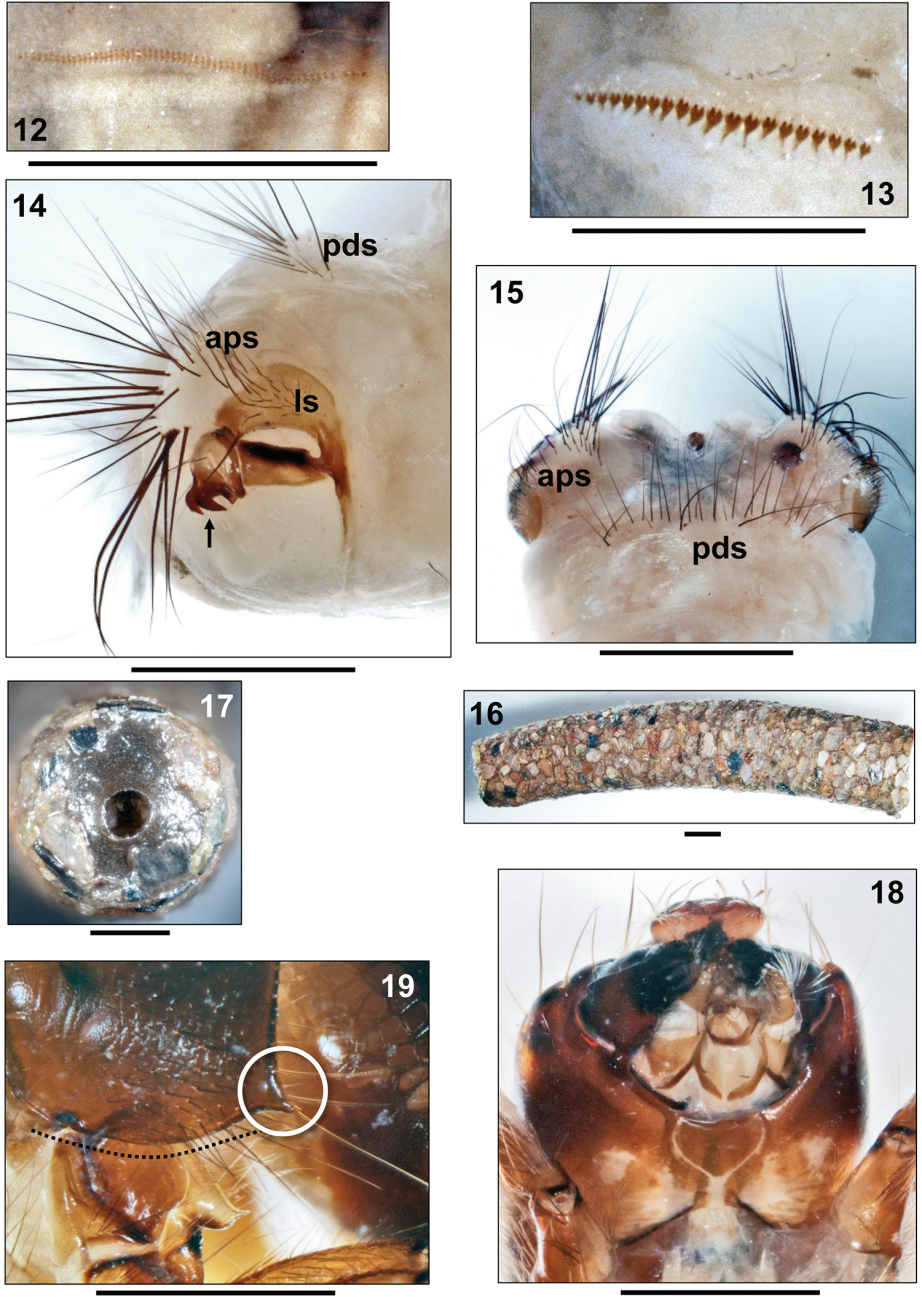
**12–17** *Sericostoma bergeri* Malicky, 1973, final instar larva **12** Abdominal segment V, posterior section, left lateral, showing row of serrate lamellae **13** Abdominal segment VIII, anterior section, left lateral, showing row of forked lamellae **14** Tip of abdomen, right lateral (aps: setae on dorsum of anal proleg; ls: setae on lateral sclerite; pds: setae on posterodorsal border of abdominal dorsum IX; arrow: anal claw) **15** Tip of abdomen, dorsal (aps: setae on dorsum of anal proleg; pds: setae on posterodorsal border of abdominal dorsum IX) **16** Larval case, right lateral **17** Larval case, foramen posterior, reduced in diameter by silk membrane **18–19** *Sericostoma herakles* Malicky, 1999, final instar larva. **18** Head, ventral view **19** Detail of pronotum (right lateral) showing conically prolonged and pointed anterolateral corner (white circle) and curved ventral outline (black dots). Scale bars: 1 mm (except **12, 13**: 0.5 mm).

**Abdomen.** Abdominal segment I with 2 flat, oblique lateral and 1 low, inconspicuous dorsal protuberances (Fig. 10); setation consisting of 1 pair of ventral *sa*1 setae (Fig. 11, arrows) and 1 lateral protuberance seta per side (Fig. 11 lp). With black comma-like marking on lateral protuberance (Fig. 10, arrow). Gills consisting of tiny single (rarely double or triple) filaments and in presegmental position only. Dorsal gills present at most from abdominal segment I to VI, ventral gills from segment II to VII and lateral gills from II to III. Lateral fringe lacking; however, with lateral rows of tiny serrate lamellae on each side of abdominal segments III to VII (Fig. 12), and with row of forked lamellae on each side of segment VIII (Fig. 13).

Dorsal sclerite of abdominal segment IX lacking, soft cuticle with 18–41 black setae of almost equal length on posterodorsal border (Figs 14, 15pds). Dorsum of each anal proleg with cluster of 35–45 black setae (Figs 14, 15 aps). Lateral sclerite of anal proleg with 28–35 black setae of varying length (Fig. 14 ls). Anal proleg claw with sharply angled crook and dorsal accessory hook (Fig. 14, arrow).

**Larval case.** Cylindrical, tapering, curved, made of flat sandgrains of approximately uniform size, neatly arranged in a puzzle-like pattern, thereby creating a rather smooth surface (Fig. 16). Case length 15.7 to 17.6 mm, anterior width 3.3 to 4.1 mm, posterior width 2.6 to 2.8 mm (n= 3). Foramen posterior partly closed by a slightly conical, translucent silken membrane with round central hole 0.61 mm in diameter (Fig. 17).

### Sericostoma herakles

Taxon classificationAnimaliaTrichopteraSericostomatidae

Malicky, 1999

#### Description of the 5^th^ instar larva.

**Diagnosis.** Pronotum with convex ventral border; anterolateral pronotal corner conically prolonged and pointed; with black comma-like marking on lateral protuberance; setal counts on abdominal dorsum IX 18–41.

**Biometry.** Body length of 5th instar larva 13.7 mm, head width 1.93 mm (n = 1). All morphological characters identical to those of *S. bergeri* except as noted below.

**Head**. Head capsule dorsally medium brown, whitish coloration on ventral section of parietalia restricted to small oval patches (Fig. 18).

**Thorax.** Pronotum with convex ventral border (Fig. 19); anterolateral pronotal corner conically prolonged and pointed (Fig. 19, white circle). Each pronotal half covered by 190-220 setae concentrated on anterior pronotal section. Sclerotized plates on mesodorsum creamish white to light brown, with oval, pale muscle attachment spots. Setal counts per central sclerite are 69–85 in anterior group and 25–30 in posterior group; lateral sclerite with 57-80 dark setae. Setal counts per metanotal sclerite are 27–35 setae in anterior group, 50–65 setae in posterior group.

**Abdomen.** Lateral sclerite of anal proleg with 35–42 black setae of varying length.

**Larval case.** Case length 15.2 mm, anterior width 3.5 mm, posterior width 2.3 mm (n= 1).

### Morphological separation of fifth instar larvae of *Sericostoma bergeri* Malicky, 1973 and *S. herakles* Malicky, 1999 from other European Trichoptera

A summary of morphological features for the identification of European caddisfly families was given by Waringer and Graf (2013). Within the framework of the Sericostomatidae key by Pitsch (1993), Wallace et al. (2003), and Waringer and Graf (2011), the larvae of the two Greek *Sericostoma* species can be separated from other species by the following features:

- pro- and mesonotum completely, metanotum incompletely sclerotized (metanotal sclerites may lack colour; Fig. 5);

- mesonotum divided into two large, central sclerites (Fig. 6cs) and two small, lateral sclerites (Fig. 6ls);

- with transportable cases (Fig. 16);

- prosternal horn lacking;

- abdominal dorsum IX unsclerotized (Fig. 15);

- abdominal segment I with one dorsal and two lateral protuberances (Fig. 10).

In the context of the Sericostomatidae larvae of European Ecoregion 6 (Hellenic western Balkan region), *Sericostoma bergeri*, and *S. herakles* can be identified by the following dichotomic key.

### Key to the final instar *Sericostoma* larvae of European Ecoregion 6 (Hellenic western Balkan)

**Table:** 

1	Pronotum with straight ventral border (Fig. 23, dotted line); abdominal dorsum IX with 18–41 setae (as in Fig. 15 pds); without black comma-like marking on lateral protuberance (as in Fig. 24, arrow)	***Schizopelex huettingeri* Malicky, 1974**
–	Pronotum with convex ventral border (Figs 4, 19, dotted curvature); setal counts on abdominal dorsum IX either 18–41 or 48–74 (Figs 15, 25); with or without black comma-like marking on lateral protuberance (Figs 20, 24 arrows)	**2**
2	Abdominal dorsum IX with 18–41 setae (Fig. 15); black comma-like marking present on lateral protuberance (Fig. 20, arrow)	**3**
–	Abdominal dorsum IX with 48–74 setae (Fig. 25); without black comma-like marking on lateral protuberance (Fig. 24, arrow)	***Oecismus monedula* (Hagen, 1859)**
3	Anterolateral corner of pronotum conically prolonged and pointed (Figs 19, 26)	***Sericostoma herakles* Malicky, 1999** (endemic of the Peloponnese) or ***Sericostoma flavicorne* Schneider,1845 / *Sericostoma personatum* (Kirby & Spence, 1826)** (not separable) (unknown from the Peloponnese so far)
–	Anterolateral corner of pronotum short and knob-like (Figs 3, 4); micro-endemic of the Greek Islands of Euboea and Andros	***Sericostoma bergeri* Malicky, 1973**

**Figures 20–26.**
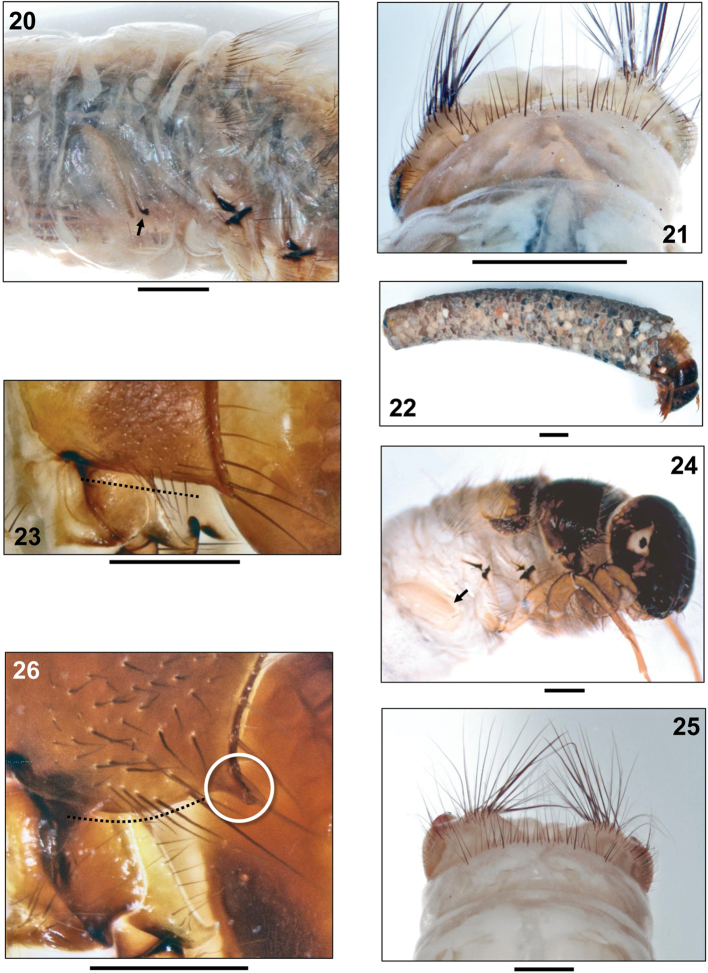
**20–22** *Sericostoma herakles* Malicky, 1999, final instar larva **20** Metanotum and abdominal segment I, right lateral (arrow: lateral protuberance with black, comma-like marking dorsally of lateral protuberance seta) **21** Tip of abdomen, dorsal **22** Larva in case, right lateral **23** *Schizopelex huettingeri* Malicky 1974, final instar larva. Pronotum, right lateral (dotted line: straight ventral margin of pronotum) **24–25** *Oecismus monedula* (Hagen 1859) **24** final instar larva, right lateral (arrow: lateral protuberance without black, comma-like marking dorsally of lateral protuberance seta) **25** Tip of abdomen, dorsal **26** *Sericostoma* sp., final instar larva. Detail of pronotum (right lateral) showing conically prolonged and pointed anterolateral corner (white circle) and curved ventral outline (black dots). Scale bars: 1 mm (except **23–26**: 0.5 mm).

### Ecology and distribution

In Europe, the Sericostomatidae fauna consists of *Cerasma cornuta* McLachlan, 1876, 6 species of *Notidobia* Stephens, 1829, 3 species of *Oecismus* McLachlan, 1876, 6 *Schizopelex* species and 18 *Sericostoma* species (García de Jalón and Vera 1978; Graf et al. 2008; Malicky 2004, 2005a, b, 2014; Ruíz-García and Ferreras-Romero 2014); of this inventory, three *Notidobia*, three *Oecismus*, one *Schizopelex* and four *Sericostoma* species have been recorded in European Ecoregion 6 (= Hellenic western Balkan). Here, the number of endemic caddisfly species is especially high: in Greece it is up to 72, yielding a proportion of 24% when compared with the overall Greek inventory of approximately 300 species. The Cyclades and Crete have the highest share of endemic species with species numbers reflecting permanent stream density; both are highest on Andros, Naxos, Ikaria and within the Ochi mountains in the south of Euboea. Indeed, on the verdant island of Andros, one third of the caddisfly fauna is endemic. Many endemics on this island have more widely distributed, close relatives within the region, e.g., *Tinodes* and *Hydropsyche* species. *Sericostoma bergeri* is such a typical micro-endemic of the Greek Islands of Euboea (Ochi mountains) and Andros. *S. bergeri* inhabits small springs and spring brooks on slate, shaded by riparian trees such as *Alnus glutinosa* (L.) Gaertn. and *Platanus orientalis* L. which release large amounts of leaf litter in the brooks. At typical habitats, water temperatures were 9.3–10.7 °C in April, 13.2–15.0 °C in May, 12.8–18.7 °C in June and 9.3–19.8 °C in October (Malicky 2014). The species is univoltine and stenochorous, peaking in emergence in May and June. In contrast to the high number of endemic species on the Greek islands, there are no significant concentrations of endemics on the Greek mainland, where most endemic species are widely spread over the mountains of central Greece or the Peloponnese (Malicky 2005b). A fine example of the latter is *Sericostoma herakles*; on the Peloponnese, this species has been mostly collected in large, calcareous mountain brooks with low annual and diurnal water temperature amplitudes (May morning water temperature: 8.1 °C, noon: 10.1 °C, evening: 9.5 °C; August evening: 14.2 °C, midnight: 13.8 °C; October morning: 12.0 °C, noon: 12.2 °C, evening: 12.0 °C [Malicky 2014]). The species is univoltine and on the wing from April to August, peaking in May; a single specimen has been collected as late as October (Malicky 2005b). The larvae of both species are detritivore-shredders, feeding on leaf litter from the woody riparian zone and aquatic vegetation, particularly mosses.

## Supplementary Material

XML Treatment for Sericostoma bergeri

XML Treatment for Sericostoma herakles
